# Genetic diversity and population structure of *Terapon jarbua* (Forskål, 1775) (Teleostei, Terapontidae) in Malaysian waters

**DOI:** 10.3897/zookeys.911.39222

**Published:** 2020-02-12

**Authors:** Shyama Sundari Devi Chanthran, Phaik-Eem Lim, Yuan Li, Te-Yu Liao, Sze-Wan Poong, Jianguo Du, Muhammad Ali Syed Hussein, Ahemad Sade, Richard Rumpet, Kar-Hoe Loh

**Affiliations:** 1 Institute of Ocean and Earth Sciences, University of Malaya, Kuala Lumpur 50603, Malaysia University of Malaya Kuala Lumpur Malaysia; 2 Institute for Advanced Studies, University of Malaya, Kuala Lumpur 50603, Malaysia Third Institute of Oceanography, Ministry of Natural Resources Xiamen China; 3 Third Institute of Oceanography, Ministry of Natural Resources, Xiamen 361005, China National Sun Yat-sen University Kaohsiung Taiwan; 4 Department of Oceanography, National Sun Yat-sen University, Kaohsiung 80424, Taiwan Ministry of Natural Resources Xiamen China; 5 Fujian Provincial Station for Field Observation and Research of Island and Coastal Zone in Zhangzhou, Xiamen 361005, China Station for Field Observation and Research of Island and Coastal Zone Xiamen China; 6 Fujian Provincial Key Laboratory of Marine Ecological Conservation and Restoration, Xiamen 361005, China Key Laboratory of Marine Ecological Conservation and Restoration Xiamen China; 7 Endangered Marine Species Research Unit, Borneo Marine Research Institute, Universiti Malaysia Sabah, Kota Kinabalu 88400, Sabah, Malaysia Universiti Malaysia Sabah Kota Kinabalu Malaysia; 8 Department of Fisheries Sabah, Kota Kinabalu 88624, Sabah, Malaysia Department of Fisheries Sabah Kota Kinabalu Malaysia; 9 Fisheries Research Institute, Sarawak, Department of Fisheries Malaysia, Kuching 93744, Sarawak, Malaysia Department of Fisheries Malaysia Kuching Malaysia

**Keywords:** COI, crescent perch, Cyt *b*, historical demography, ikan mengkerong, Pleistocene

## Abstract

A background study is important for the conservation and stock management of a species. *Terapon jarbua* is a coastal Indo-Pacific species, sourced for human consumption. This study examined 134 samples from the central west and east coasts of Peninsular (West) Malaysia and East Malaysia. A 1446-bp concatenated dataset of mtDNA COI and Cyt *b* sequences was used in this study and 83 haplotypes were identified, of which 79 are unique haplotypes and four are shared haplotypes. Populations of *T. jarbua* in Malaysia are genetically heterogenous as shown by the high level of haplotype diversity ranging from 0.9167–0.9952, low nucleotide diversity ranging from 0.0288–0.3434, and high F_ST_ values (within population genetic variation). Population genetic structuring is not distinct as shown by the shared haplotypes between geographic populations and mixtures of haplotypes from different populations within the same genetic cluster. The gene flow patterns and population structuring observed among these regions are likely attributed to geographical distance, past historical events, allopatric speciation, dispersal ability and water currents. For instance, the mixture of haplotypes revealed an extraordinary migration ability of *T. jarbua* (>1200 km) via ancient river connectivity. The negative overall value of the neutrality test and a non-significant mismatch distribution are consistent with demographic expansion(s) in the past. The median-joining network concurred with the maximum likelihood haplotype tree with three major clades resolved. The scarcity of information on this species is an obstacle for future management and conservation purposes. Hence, this study aims to contribute information on the population structure, genetic diversity, and historical demography of *T. jarbua* in Malaysia.

## Introduction

A population’s genetic structure describes the total genetic diversity in the population, which is shaped by several factors, including the life history, geographical barriers, gene flow, selection and bottlenecks (Wright 1931; Slatkin 1987; Charlesworth 2009; Liu et al. 2019). The patterns of genetic diversity and population structure provide information on the life histories, demography, reproduction and ecology of a species. This information is important for a population’s sustainability by implementation of appropriate conservation and management strategies (Okumuş and Çiftci 2003; Zaya et al. 2017).

*Terapon jarbua* (Forskål, 1775) is a medium-sized fish commonly known as crescent perch, and it is locally known as “ikan mengkerong” in Malaysia (Department of Fisheries Malaysia 2009). This species is classified under the class Actinopterygii, order Perciformes and family Terapontidae (Froese and Pauly 2018). Although it is primarily a marine species, it has also been found in coastal areas, estuaries, freshwaters and in some coastal lagoons (Rao et al. 2000). It is categorized as a catadromous fish, in which the adults spawn in deeper saltwater while the juveniles move to the shallow sandy bottom area near the river mouths. According to Lavergne et al. (2012), the pelagic larval phase of this species is about 25 days. *Terapon jarbua* is classified as least concern (LC) under the IUCN Red List due to its widespread distribution with no known threats (Dahanukar et al. 2017). The native distributional ranges of the crescent grunters include Australia, Bangladesh, Cambodia, China, India, Indonesia, Japan, Malaysia (Du et al. 2019; Shyama et al. 2020), Mediterranean (Golani and Appelbaum-Golani 2010), Myanmar, Philippines, Red Sea, Sri Lanka and Taiwan (Froese and Pauly 2018).

Existing reports on *T. jarbua* are generally limited to their morphometry (e.g., length-weight relationship) and reproductive biology (Miu et al. 1990; Nandikeswari et al. 2014; Musarrat and Masood 2015) while the information on their genetic diversity and population structures is relatively little. Lavergne et al. (2012) and Liu et al. (2015) reported on the population genetic diversity of *T. jarbua* in the Gulf of Aden, Yemen and Taiwanese waters, respectively, using cytochrome *c* oxidase subunit I (COI), cytochrome *b* (Cyt *b*) and microsatellites molecular markers. Also, a phylogeographic survey of *T. jarbua* along with other reef fauna of the western Indian Ocean was reported by Borsa et al. (2016) using COI gene sequences. Mitochondrial DNA (mtDNA) has been widely utilized as the marker of choice to examine the genetic diversity and population structure of marine fishes due to its strict maternal inheritance, rapid mutation rates and the absence of recombination in most species (Whitehead et al. 2003; Dowling et al. 2008; Song et al. 2013).

**Figure 1.**
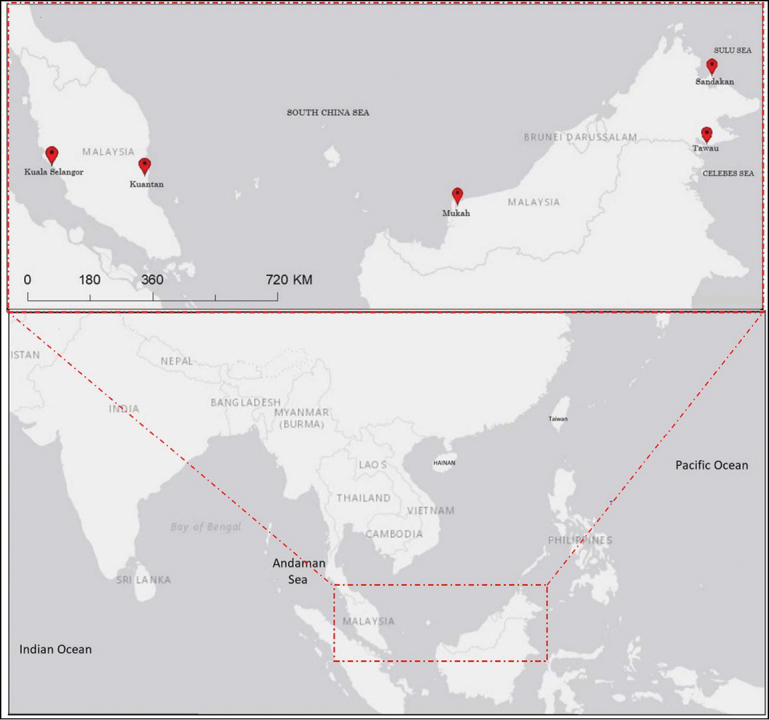
Sampling localities from East (Sandakan and Tawau, Sabah & Mukah, Sarawak) and West (Peninsula) Malaysia (Kuala Selangor, Selangor and Kuantan, Pahang).

The main focus of the current study is on the Malaysian populations: Peninsular (West) Malaysia and East Malaysia (Sabah and Sarawak) which are located in the tropical Indo-west Pacific region (Fig. 1). These two land masses are about 1200 km apart, separated by the south-western portion of the South China Sea. In this study COI and Cyt *b* were used as molecular markers to examine the level of gene flow, population genetic differentiation and the historical demography of *T. jarbua* populations in Malaysia. To the best of our knowledge, there is no documented report on the population genetics of *T. jarbua* in Malaysia to date. Hence, this study aims to provide a documented background report as well as to fill the information gap for *T. jarbua* in this region. Homologous COI+ Cyt *b* sequences of four regional representatives of this species from India, Taiwan, Hainan and the Philippines were included in the analysis to provide a wider coverage of the species’ natural distribution.

## Materials and methods

### Sampling

Sampling around major landing sites and local markets was conducted in both East and Peninsular Malaysia where 134 samples of various sizes were collected randomly from five wild populations of *T. jarbua*. Populations were provisionally divided into five groups according to region: 1) Kuala Selangor (KS, *N* = 31) of west Peninsular which is surrounded by the Straits of Malacca; 2) Kuantan, Pahang (KN, *N* = 30) of east Peninsular which is adjacent to the South China Sea; 3) Mukah, Sarawak (MH, *N* = 21) of East Malaysia which is surrounded by the South China Sea; 4) Sandakan (SN, *N* = 28) and 5) Tawau (TW, *N* = 24) of East Malaysia which are surrounded by the Sulu Sea and the Celebes Sea, respectively (Fig. 1). Samples were collected within the period of April 2015 to August 2018. Approximately 20 mg of muscle tissue from each fish sample was removed and immediately preserved in 95% ethanol and stored at -20°C until genetic analysis was performed.

### DNA extraction, PCR amplification and DNA sequencing

Genomic DNA was extracted using 10% Chelex Resin following the protocol of Hyde et al. (2005). Approximately 680 bp of the COI-5’ gene was amplified using the FishF1 or FishF2 forward primers and FishR1 or FishR2 reverse primer pairs (Ward et al. 2005). Polymerase Chain Reaction (PCR) amplification of approximately 1000 bp from the 5’-end of the Cyt *b* gene was performed using the primer pairs Glu31 and Thr33 (Liu et al. 2015) and internal primers: Glu231 (5’-CTT ACA GGC CTC TTT CTG GCC AT- 3’) and Thr233 (5’- TTT GAG CTA CTA ATG CAG TAT- 3’) were designed for this study.

PCR was performed using a Mastercycler epgradient S thermalcycler (Eppendorf, Hamburg, Germany) and 25μl reaction mixtures consisting of 12.5μl exTEN 2X PCR master mix (1^st^ BASE, Selangor, Malaysia), 9.5 μl of sterile distilled water, 1μl each of forward and reverse primers, and 1μl of DNA template. PCR cycling conditions were as follow: initial denaturation for 1 min at 96°C, 36 cycles of denaturation at 95°C for 30 s, annealing for 30 s at 44°C (CO1) or 48°C (Cyt *b*), elongation for 1 min at 72°C, and final elongation for 10 min at 72°C. The amplicons were checked for correct length via electrophoresis on a 1% agarose gel (90V for 25 min). PCR products were sent to Apical Scientific Sdn. Bhd. (Selangor, Malaysia) for purification and DNA sequencing.

### Sequence analysis

Multiple sequence alignment was first performed separately for each gene region using the CLUSTAL X (Thompson et al. 1994) program implemented in BIOEDIT ver. 7.0.5 (Hall et al. 2011). The sequences were subsequently trimmed and aligned manually prior to concatenation of COI and Cyt *b* sequences. Analyses performed in this study were based on the final truncated length of 1446-bp concatenated sequences. All haplotype sequences were deposited in Genbank under the accession numbers MN529663–MN52993.

Unique haplotypes were quantified and the genetic diversity, nucleotide diversity, and pairwise distance were calculated using DNASP v. 4.0 (Rozas et al. 2003). The level of gene flow among populations (Nm) based on Hudson et al. (1992) was also calculated in DNASP v. 4.0. Analysis of molecular variance (AMOVA) was performed using ARLEQUIN v.3.5 (Excoffier and Lischer 2010) for the four, hypothetical, region-based groupings (Selangor, Pahang, Sabah and Sarawak) to investigate the partition of genetic variation among regions (F_CT_), among populations within regions (F_SC_), and within populations (F_ST_). The significance of the F-statistics for population comparisons was assessed using 1000 permutations. The Tamura Nei plus gamma rate model (TN93+G) was selected by MEGA v. 7.0 (Kumar et al. 2016) as the best-fitting substitution model based on the Bayesian information criterion. A Maximum Likelihood (ML) tree was reconstructed in MEGA 7.0 to show the level of divergence and relationships among haplotypes of *T. jarbua*. The confidence level at each node was assessed by 1000 bootstrap replications. This tree was compared against the median-joining network generated using the program’s default settings of NETWORK 4.5.0.2 (Bandelt et al. 1999).

In addition, a neutrality test of the pairwise differences among all populations was performed to infer historical demographic and deviation of sequence variation from evolutionary neutrality. Deviations from neutrality were evaluated using Fu’s Fs (Fu 1997) and Tajima’s *D* (Tajima 1989) via DNASP. Statistical tests and confidence intervals for *D* and F’s were based on a coalescent simulation algorithm. A large negative value of Fu’s Fs or the Tajima’s *D* rejecting the null hypothesis of neutrality indicates population expansion(s). The demographic changes were also examined using the mismatch distribution analysis (Rogers and Harpending 1992) in ARLEQUIN with 1000 permutations. The Harpending’s raggedness index (Harpending et al. 1993) and the sum of squared deviations (SSD) between observed and expected mismatch for each of the populations under the model of constant population size were analyzed according to Schneider and Excoffier (1999). This method quantifies the smoothness of the observed mismatch distribution and a non-significant result indicates an expanding population. The spatial expansion hypothesis (both raggedness index and SSD) was tested using a parametric bootstrap approach with 1000 replicates.

## Results

### Genetic diversity

The 1446 bp concatenated COI (631 bp) and Cyt *b* (815 bp) sequences were analyzed for 134 individuals obtained in five different locations (Fig. 1) from East Malaysia and Peninsular Malaysia. The nucleotide composition was 23.0% adenine, 28.9% thymine, 32.0% cytosine and 16.1% guanine. The higher A+T content (51.9%) compared to G+C content (48.1%), is common among fishes (Appendix 1). There were 202 polymorphic sites of which 31 (15.4%) were singleton variable sites, and 171 (84.6%) were parsimony informative (Appendix 2). A total of 149 mutations with 66 transitions, 8 transversions and 75 substitutions were found within the dataset (data not shown).

A total of 83 putative haplotypes were derived from the 134 individuals sequenced with 79 of them being unique haplotypes (95.18%) and four were shared haplotypes (4.82%). The dominant haplotype of Malaysian populations is Hap5 (KS, KN, TW, SN, MH, TAI) while other shared haplotypes are Hap3 (KS, KN, TW, MH, IND), Hap8 (KS and KN) and Hap45 (TW and SN). The population from KS recorded the highest total number of haplotypes (22) of which 19 were unique haplotypes, while Tawau recorded the lowest number of haplotypes (15) with 12 unique haplotypes. The nucleotide diversity (*π*) of *T. jarbua* populations in this study ranged from 0.0288 ±0.0158 (mean ±SD) to 0.3434 ±0.1722 while haplotype diversity (*h*) ranged from 0.9167 ±0.0482 to 0.9952 ±0.0165 (Table 1). The MH population recorded the highest *π* and *h.* A high *h* and low *π* indicate that the populations studied were moderate in genetic diversity.

**Table 1.** Information and molecular indices of *T. jarbua*. N, number of samples; NH, number of haplotypes; NUH, number of unique haplotypes; *h*, haplotype diversity; *π*, nucleotide diversity; *k*, average number of pairwise differences.

**ID**	**Populations**	**N**	**NH**	**NUH**	***h***	***π***	***k***
KS	Kuala Selangor, Selangor	31	22	19	0.9828 ±0.0135	0.1817 ±0.0904	36.5161 ±16.3285
KN	Kuantan, Pahang	30	19	16	0.9678 ±0.0208	0.0288 ±0.0158	5.7885 ±2.8485
MH	Mukah, Sarawak	21	16	14	0.9952 ±0.0165	0.3434 ±0.1722	69.0238 ±31.0005
SN	Sandakan, Sabah	28	20	18	0.9577 ±0.0262	0.0487 ±0.0256	9.7810 ±4.6212
TW	Tawau, Sabah	24	15	12	0.9167 ±0.0482	0.0514 ±0.0271	10.3333 ±4.8857
Total		134	83	79	0.9820 ±0.0050	0.0248 ±0.0031	35.8653 ±12.2638

### Genetic structure

**Figure 2.**
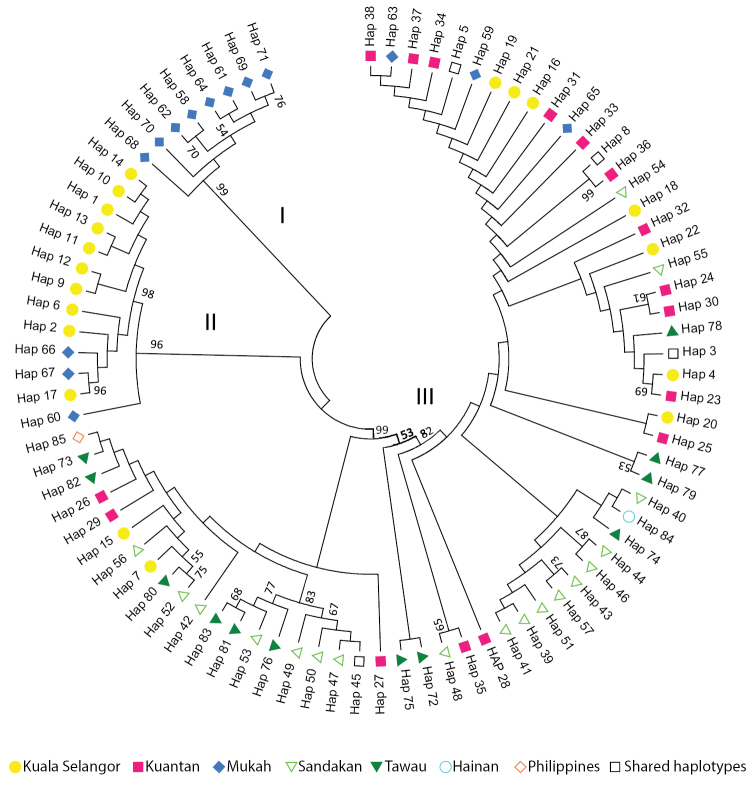
Maximum likelihood haplotype tree reconstructed based on the concatenated mtDNA dataset. The bootstrap values higher than 50% are shown near the nodes.

A ML tree was reconstructed based on the 83 haplotypes of this study and four COI + Cyt *b* sequences from Hainan, Taiwan, India and Philippines which were downloaded from National Center for Biotechnology Information (NCBI) (Appendix 3). The mtDNA concatenated dataset defined the haplotypes into three major clades with no significant clusters corresponding to sampling localities (Fig. 2). Apart from Clade I which consists of eight haplotypes solely from Sarawak, the other four clades include mixtures of haplotypes from various localities without any obvious geographical structuring among them. Clade II consists of haplotypes from MH and KS populations while Clade III consists of the haplotype from the Philippines and haplotypes from Malaysia except MH. Clade III is the most geographically inclusive with haplotypes from India, Hainan, Taiwan, Philippines and all five Malaysian populations.

**Figure 3.**
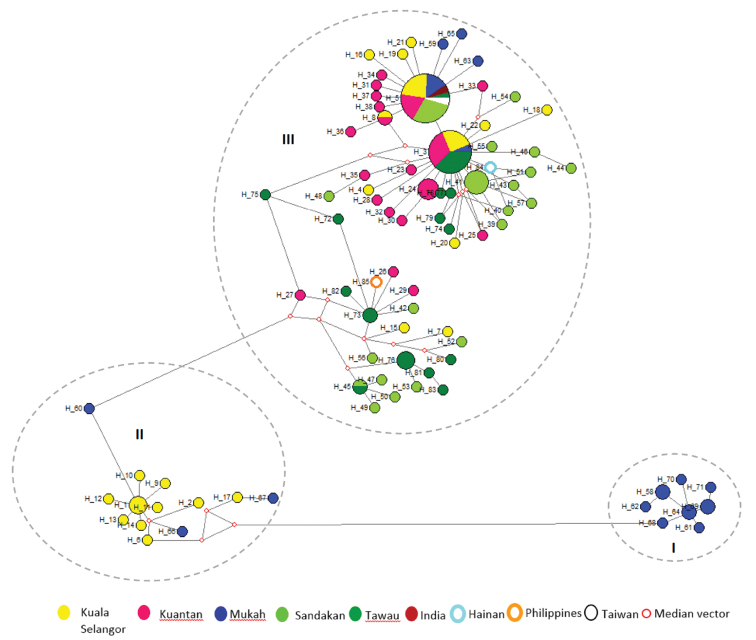
Haplotypes median-joining network corresponding to the ML tree with three observed clusters. The star-like profile observed in cluster III indicates the presence of sudden expansion.

The general topology of the median-joining network (Fig. 3) corresponded with the ML tree (Fig. 2) with three major clusters identified. In the network presented, shared haplotypes occupy the central area, while the unique haplotypes branched out from the center. This formation provides a star-like profile, which indicates population expansions. Hap3 and Hap5 are the dominant haplotypes in cluster III. The distribution frequency of all the 83 haplotypes in *T. jarbua* populations is presented in Appendix 4. Hap5 recorded the highest distribution frequency with 21 individuals. It is the only common haplotype shared between all five populations from Malaysia and the representative from Taiwan. Hap3 with 16 individuals was found in India and in all locations sampled in Malaysia except for Sandakan.

Pairwise F_ST_ comparisons between populations in Malaysia were significant at the 95% confidence level except for the comparison between TW and SN (Table 2). Populations of MH and KN showed the greatest pairwise differentiation (F_ST_ = 0.5353; *p* < 0.05) while SN and TW showed the least differentiation (F_ST_ = 0.0452; *p* > 0.05). The pairwise nucleotide divergence among populations (Table 3) showed the same trend as the F_ST_ values and was not correlated with geographical distance. The overall gene flow (Nm) estimated among populations was low at 0.82. The sequence divergence was calculated using the Kimura two parameter (K2P) distance model for both genes (Table 3). The greatest genetic differences (COI: 0.019 and Cyt *b*: 0.029) were observed between MH-KN, MH-SN and MH-TW. The *T. jarbua* populations displayed a low level of conspecific divergence within 2% (COI).

The genetic structure of the *T. jarbua* populations analysed by AMOVA showed little (39.52%) genetic differentiation among regions but high (62.13%) variation within populations (Table 4). This indicates that the populations were not genetically differentiated among regions and the genetic variation was mainly from within the population level. There is essentially no genetic structuring (-0.14% variation) among populations within region.

**Table 2.** Pairwise F_ST_ (below diagonal) and exact *P*-values (above diagonal) among five populations of *T. jarbua* based on 1000 permutations of the sequence data set. Numbers in bold represent the highest and lowest value. *Significant at *p* <0.05 by the permutation test. Overall gene flow (N_m_) is 0.82.

**Populations**	**KS**	**KN**	**MH**	**SN**	**TW**
KS	-	0.0000*	0.0000*	0.0000*	0.0000*
KN	0.2965	-	0.0000*	0.0270*	0.0090*
MH	0.3310	**0.5353**	-	0.0000*	0.0000*
SN	0.2681	0.0702	0.5038	-	0.0541
TW	0.2633	0.1773	0.4844	**0.0452**	-

**Table 3.** Net between-group mean distances using Kimura-2-parameter (K2P) model.

	**Populations**	**KS**	**KN**	**SN**	**MH**	**TW**
COI	KS	-				
	KN	0.003	-			
	SN	0.003	0.000	-		
	MH	0.015	0.019	0.019	-	
	TW	0.004	0.001	0.000	0.019	-
Cyt *b*	KS	-				
	KN	0.008	-			
	SN	0.008	0.001	-		
	MH	0.018	0.029	0.029	-	
	TW	0.008	0.001	0.000	0.029	-

**Table 4.** AMOVA of *T. jarbua* samples based on mtDNA sequences.

**Source of variation**	**Sum of squares**	**Percentage of variation**	**F statistic**	***P***
Among region (F_CT_)	800.958	39.52	0.3952	0.1896 ± 0.0134
Among populations within region (F_SC_)	11.1610	-0.14	-0.0033	0.0831 ± 0.0082
Within populations (F_ST_)	1476.92	62.13	0.3952	0.0000 ± 0.0000

### Historical demography

The overall Tajima’s *D* value was negative with an insignificant *p*-value, indicating deviation from evolutionary neutrality. Similarly, the Fu’s Fs test which is based on the distribution of haplotypes, revealed negative but significant *p*-values for all five populations studied, indicating an excess of rare haplotypes or rare mutations in the population compared to what is expected under a neutral model of evolution. Following the results of Fu’s Fs test, the hypothesis of neutral evolution was rejected.

**Figure 4.**
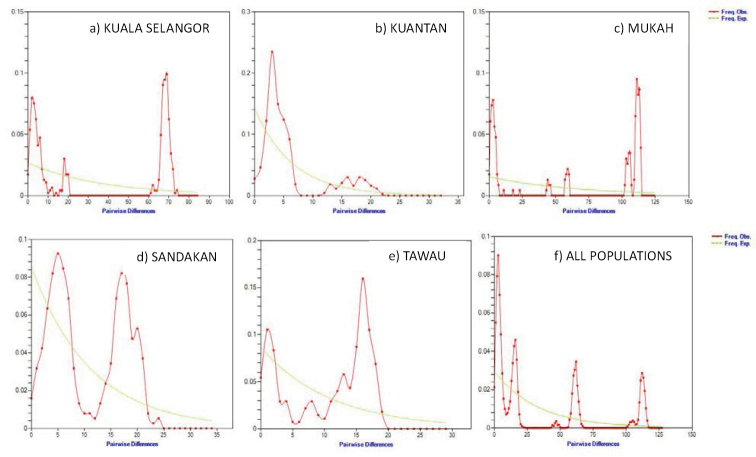
Pairwise number of difference (mismatch distribution) analysis was conducted using the constant population size model to observe the population size changes. The observed frequencies were represented by red dotted line. The frequency expected under the hypothesis of population expansion model was depicted by continuous green line. **a** Kuala Selangor **b** Kuantan **c** Mukah **d** Sandakan **e** Tawau **f** all populations.

In the present study, all populations demonstrated bimodal and ragged shaped patterns which points to the population having remained largely constant in size and that the lineage was widespread (Rogers and Harpending 1992). The scatterplot of the bimodal illustrations is shown in Figure 4. The results of mismatch distribution are contradictory to the results of the neutrality analysis. Hence, to further test the validity of the neutrality test results, we calculated the raggedness index and SSD under the demographic expansion model as shown in Table 5. *P*-values of SSD between the observed and expected mismatch distributions were all statistically insignificant (*p* > 0.10), indicating the presence of non-equilibrium and a population expansion event in *T. jarbua*. Besides that, the studied populations showed non-significant raggedness index (*p* > 0.10) indicating the data has relatively good fit to a model of population expansion (Harpending 1994). A ragged distribution suggests that the lineage was widespread (Excoffier et al. 1992; Rogers and Harpending 1992; Rogers 1995).

**Table 5.** Parameter estimates of neutrality tests (Tajima’s *D* statistic and Fu’s Fs) and mismatch distribution (sum of squares deviation (SSD) and r = raggedness index) for each population. Significance (*p < 0.10) was determined using coalescent simulations.

	**Neutrality test**		**Mismatch distribution**		
	**Tajima’s *D***	**Fu’s F_S_**	**SSD**	**r**	**Curve**
KS	1.4817	-1.252	0.0296	0.0106	Bimodal
KN	-1.2595	-11.560	0.0113	0.0333	Bimodal
SN	-0.4265	-6.153	0.0301	0.0160	Bimodal
TW	1.0793	-2.075	0.0236	0.0266	Bimodal
MH	2.1863*	-1.093*	0.0288	0.0142	Bimodal
Total	-0.3132	-24.885*	0.0247	0.0201	Bimodal

## Discussion

### Genetic diversity

Species identification was confirmed by morphological observation and DNA sequence data in which the intraspecific COI divergence was within the 2% threshold value (Hebert et al. 2003; Ward 2009; Srivathsan and Meier 2012). In general, a relatively high haplotype diversity (0.92–0.99) and low nucleotide diversity (0.03–0.34) were observed for the populations in this study. The combination of high haplotype diversity and low nucleotide diversity is common in pelagic marine fishes (Liu et al. 2015). This is likely due to rapid demographic expansion from a small effective population size, assuming there is sufficient time for the number of haplotypes to increase through mutation but insufficient for accumulation of large sequence differences (Grant and Bowen 1998; Avise 2000; Lowe et al. 2004). The inference of population expansion is further supported by the star-like patterns in Figure 3. Our results agree with an earlier study of *T. jarbua* populations from the Taiwanese waters which exhibited similarly high population haplotype diversity ranging from 0.86 to 1.00 as inferred from COI and Cyt *b* sequence data (Liu et al. 2015). In a study on *T. jarbua* populations from the Gulf of Aden, Lavergne et al. (2014) reported similarly high genetic diversities ranging from 0.216 to 0.698. The high number of haplotypes (83 haplotypes) in the present study is likely due to the high mutation rate of the mtDNA genes. Most of the haplotypes are unique to its region which may indicate the presence of different founding populations in the studied localities (Teixeira et al. 2011). The haplotype diversity of a relatively rapid evolving genome within a population often approaches 1.0 as many individuals will tend to have unique haplotypes (Freeland et al. 2011).

### Genetic structure

A population’s genetic structure is affected by genetic drift, local adaptation, and gene flow. In a marine environment, the development of population structure is greatly influenced by factors that affect dispersal, such as ocean currents, historical variance, and geographic distance coupled with differences in dispersal ability and habitat discontinuity (Saarman et al. 2010). Population structure inferred from mtDNA markers displays less genetic divergence in the pelagic and moderately pelagic species due to their potential to undertake long-distance migrations in oceanic waters (Jaafar 2014).

The haplotype tree (Fig. 2) revealed three major lineages but geographic structuring among the five populations is not distinct. In general, haplotypes specific to certain geographic regions did not form monophyletic groups, but appeared to be randomly distributed across the haplotype tree. Hap5 and Hap3 which recorded the highest distribution frequency are likely the ancestral haplotypes among the populations sampled. Recent haplotypes were evolved directly or indirectly from the ancestral haplotypes. The existence of two ancestral points indicates that *T. jarbua* in Malaysia probably exist from two different sources. According to the coalescent theory, common haplotypes at the center of a network are inferred to be ancestral, while tip haplotypes at the periphery are derived or descendant from ancestral haplotypes (Akib et al. 2015). The occurrence of star-like patterns radiating from these major haplotypes suggests that *T. jarbua* populations have undergone significant population size expansions in the relatively recent past (Forster et al. 2001; Akib et al. 2015).

F_ST_ values are often used to infer gene flow, in which a lower F_ST_ value indicates low genetic divergence and higher gene flow. F_ST_ values below 0.05, as observed between SN and TW populations, indicate negligible genetic divergence, probably due to active exchange of genetic material between populations through breeding. Furthermore, the pairwise divergence between these populations is not statistically significant. According to Wright (1965), F_ST_ of 0–0.05 is described as little differentiation, 0.05–0.15 as moderate differentiation, 0.15–0.25 as great differentiation and values greater than 0.25 as very great differentiation. All populations studied showed moderate to very great pairwise differentiation except for TW-SN. The overall gene flow recorded was rather low (Nm = 0.82) which suggests limited genetic connectivity among the five populations.

Populations from the same region, i.e., TW and SN of Sabah, were the least genetically variable (Tables 2 and 3), which is likely due to the close geographical distance between these populations. The theory of fish migration across adjacent drainage systems due to flooding, which follows the one-dimensional stepping stone model that allows migration to adjacent population (Song et al. 2013), may apply in the case of *T. jarbua*. The significantly higher genetic differentiation between populations of KS-SN (pairwise F_ST_ = 0.2681) compared to KN-SN (pairwise F_ST_ = 0.0702) may be attributed to distance and physical barrier. Some genetic exchange can be expected since the Straits of Malacca connects the Andaman Sea and South China Sea via the narrow Tebrau strait. It is likely that mixing between the two bodies of water is very limited which supports the F_ST_ value obtained. However, the higher genetic variation between populations of MH-SN (pairwise F_ST_ = 0.5038) as compared to MH-KS (pairwise F_ST_ = 0.3310) implies that geographical distance is not the only driving factor of genetic variation among populations of *T. jarbua* in the Malaysian waters, similar to the results observed in the wider Gulf of Aden (Lavergne et al. 2014) where populations of adjacent locations showed low genetic connectivity despite the absence of a geographic barrier. Populations bordering a common origin such as the South China Sea (KN, MH, TW, and SN) may have evolved independently of each other over time, but there might have been insufficient time for genetic divergence to accumulate in these populations.

Another interesting finding of this study is the occurrence of shared haplotype between the populations from Peninsular and East Malaysia, India, Hainan, Philippines and Taiwan. Common haplotypes between localities and mixed haplotypes of different lineages in some populations in the current study can be explained by the biogeographical history of Southeast Asia (historically known as the Sundaland). Southeast Asia is believed to have experienced simultaneous glaciation and consequent deglaciation along with its associated decrease and increase of seawater levels during the Pleistocene period, which greatly influenced continental and oceanic configuration (Voris 2000). The shared haplotypes between Malaysian populations and those from as far as India suggests that the range of population expansion after glacial retreat was not restricted to the South China Sea but also extended into the Indian Ocean (Liu et al. 2015). Lavergne et al. (2014) also reported high connectivity between populations in the Gulf of Aden and South China Sea due to the unique sharing of COI haplotypes between both regions. The haplotype sharing and their consequent gene flow may also be attributed to breeding migration, mutation, pelagic larvae, and sharing of common ancestors (Frankham 1996).

The MH population is the most genetically distinct with the highest between-group mean distances, haplotype and nucleotide diversity among the five populations. Geographical isolation of allopatric populations restricts gene flow between two populations, which in turn allows the evolution of a genome adapted to local condition (Hall 1993). Cluster I (MH) is estimated to form after the separation of Peninsular Malaysia from the Borneo Island due to the rise in the depth of the Sunda River between 40 to 100 m. This gradual separation was suspected to have caused accumulative genetic drift. According to Halliday (1993), genetic drift is likely to occur, particularly in small populations that are isolated from the main population and it may become the major source of genetic variation between some populations.

Among the four populations, MH is genetically closest to KS. Geological evidence suggests that the river systems of Sarawak were historically interconnected with most major river systems of Peninsular Malaysia via the Sunda River during Pleistocene glaciation (about 10000 years ago), thus allowing gene flow among these drainages (Kamarudin and Esa 2009). Gene flow from populations in the Straits of Malacca to those in Sarawak has been reported in several studies including Ryan and Esa (2006), Azhar and Hassan (2015), Samani et al. (2016) and Lau et al. (2018). Meanwhile, populations of SN-KN, which are separated by the South China Sea, showed high genetic connectivity (pairwise F_ST_ = 0.0702). This could probably be explained by the high migration ability of *T. jarbua* (>1000 km, Liu et al. 2015), human-mediated transfer through ballast waters (Liu et al. 2019) or past glaciation events. Furthermore, the high similarity in the sequence data (Table 3) perhaps indicates remnants of identical haplotypes from both populations, and that they were essentially similar at one time before the separation (Inger and Chin 2002). Eventually, sea level rise during the last Pleistocene period caused Borneo to be separated from mainland Asia (Peninsular Malaysia), which we suggest, resulted in shelf submergence and subsequent genetic differentiation between grunters from KN and SN. Pleistocene sea level fluctuations could also explain the incomplete divergence of grunters between East and Peninsular Malaysia. Similar evidence of a close genetic relationship between fishes of Borneo and mainland Asia in relation to their biogeographical history was discussed by several other authors (Pin et al. 2001; Nadiatul et al. 2011; Tan et al. 2012; Song et al. 2013).

### Historical demography

Historical demographic expansions were determined by analysing the frequency distributions of pairwise differences between sequences (Rogers and Harpending 1992; Ray et al. 2003; Excoffier 2004). Neutrality tests with Tajima’s *D* and Fu’s Fs statistics estimate the deviation from neutrality, which is based on the expectation of a constant population size at mutation-drift equilibrium. Here, a negative Tajima’s *D* signifies an excess of low frequency polymorphisms relative to expectation, indicating population size expansion or positive selection (Tajima 1983). The negative and significant Fu’s Fs statistical value provides strong evidence for past population expansion, and rule out the possibility of genetic hitching or background selection, and evolutionary forces that produce a pattern similar to population expansion (Fu and Li 1993; Fu 1997; Okello et al. 2005). The *T. jarbua* populations displayed a genetic pattern typical of a population that has undergone a recent population expansion due to its two common haplotypes (Hap3 and Hap5) present across the range while the rest of the haplotypes are unique. The range expansion was a recent phenomenon and may not have achieved the migration-drift equilibrium, as shown by the lack of phylogeographical structure. Neutrality test statistics were in overall negatively significant and not consistent with a population at drift-mutation equilibrium.

The mismatch distribution is generally displayed as a multimodal pattern for populations showing demographic equilibrium. In contrast, a unimodal pattern depicts populations which have experienced recent demographic expansion (Rogers and Harpending 1992). In the results, all localities presented a multimodal pattern proving recent expansion. The hypothesis that the observed data fit the sudden expansion model was tested using the SSD and the raggedness index. Here, non-significant values for SSD signifies that the observed data do not deviate from that expected under the model of expansion. Non-significant raggedness index also indicates population expansion. Our observations of non-significant values in goodness-of-fit distribution for all populations suggest that population expansion occurred recently (Rogers and Harpending 1992).

## Conclusion

To summarize, we found 1) high haplotype diversity but low nucleotide diversity among *T. jarbua* populations in Malaysia; 2) significant results suggesting population expansion of *T. jarbua* in this region; 3) despite the three genetic clusters observed in the haplotype tree and median-joining network, no obvious population structuring was detected among geographically distinct populations. Common haplotypes among populations and haplotypes from several populations in each genetic cluster indicate high genetic connectivity among the populations. This study assesses the genetic diversity and population structure of *T. jarbua* in Malaysia for appropriate conservation and management strategies. Conservation of crescent grunter at its natural variation level is required as it forms a diverse group of taxa with 83 haplotypes distributed across Malaysia. The haplotype composition surveyed in the present study may provide a baseline for future comparisons to monitor the temporal variability of haplotype frequency and population structure. This study also has indirectly revealed the dispersal power of *T. jarbua* through its high mobility and rapid adaptability to a newly colonized area. Further studies can be conducted using larger sample size and temporal replicates, samples collected from other areas of geographical distributions, and sequence data from other mtDNA genes or information based on nuclear DNA. This research contributed useful data for future large scale biogeographical and taxonomic studies of this species.

## Animal ethics

The fish species that was employed in this study is not categorized as endangered species under the IUCN list and all the samples were collected from fish markets and landing sites.

## Appendix 1

**Table A1.** Percentage of nucleotide composition based on populations.

**Nucleotide composition (%)**																		
**ID**	**COI**						**Cyt b**						**Combine**					
	**A**	**T**	**C**	**G**	**A + T**	**C + G**	**A**	**T**	**C**	**G**	**A + T**	**C + G**	**A**	**T**	**C**	**G**	**A+T**	**C+G**
KS	22.4	28.8	30.9	17.9	51.2	48.8	23.5	28.7	32.9	14.9	52.2	47.8	22.9	28.9	32.0	16.2	51.8	48.2
KN	22.6	28.9	30.7	17.8	51.5	48.5	23.5	28.7	32.8	14.9	52.2	47.7	23.0	29.0	31.9	16.1	52.0	48.0
MH	22.5	28.4	31.4	17.8	50.9	49.2	23.7	28.4	32.9	15.0	52.1	47.9	23.0	28.6	32.2	16.2	51.6	48.4
SN	22.6	28.9	30.7	17.8	51.5	48.5	23.5	28.7	32.8	14.9	52.2	47.7	23.0	29.0	31.9	16.1	52.0	48.0
TW	22.6	28.9	30.8	17.8	51.5	48.6	23.5	28.7	32.9	14.9	52.2	47.8	23.0	28.9	32.0	16.1	51.9	48.1
Total	22.2	29.2	30.9	17.7	51.4	48.6	23.6	28.7	32.9	14.9	52.3	47.8	23.0	28.9	32.0	16.1	51.9	48.1
HAI	22.5	28.9	30.7	17.8	51.4	48.5	23.4	28.8	32.9	14.8	52.2	47.7	23.1	28.9	32.0	16.1	52.0	48.1
IND	22.3	29.5	30.5	17.7	51.8	48.2	23.6	28.8	32.8	14.8	52.4	47.6	22.9	29.7	31.6	15.8	52.6	47.4
PHI	22.9	28.9	30.7	17.5	51.8	48.2	23.4	28.6	32.9	15.1	52.0	48.0	23.2	28.7	32.0	16.1	51.9	48.1
TAI	22.5	28.9	30.7	17.8	51.4	48.5	23.6	28.7	32.9	14.8	52.3	47.7	23.1	28.8	32.0	16.1	51.9	48.1

## Appendix 2

**Table A2.** Polymorphic site analysis based on COI, Cyt *b* and combined gene. C, conserved site; V, variable site; Pi, parsimony informative sites; S, singleton sites.

**ID**	**COI (631 bp)**				**Cyt *b* (815 bp)**				**Combine (1446 bp)**			
	**C**	**V**	**Pi**	**S**	**C**	**V**	**Pi**	**S**	**C**	**V**	**Pi**	**S**
KS	602	29	20	9	742	73	53	20	1344	102	73	29
KN	618	13	6	7	793	22	14	8	1411	35	20	15
MH	575	56	54	2	716	99	95	4	1291	155	149	6
SN	617	14	7	7	787	28	20	8	1404	42	27	15
TW	619	12	9	3	798	17	14	3	1417	29	23	6
Total	557	74	60	14	687	128	111	17	1244	202	171	31

## Appendix 3

**Table A3.** Sequence data used in this study. All data were downloaded from NCBI.

**Location**	**Accession number**		**Publication**
	**COI**	**Cyt *b***	
Hainan	NC027281	NC027281	Wu et al. 2016
Taiwan	KP204162	KP152133	Liu et al. 2015
Kochi, India	KC774674	KC774717	Lenka et al. 2014 (Unpublished)
Philippines	KF999840	KF999856	Canoy and Quilang 2015 (Unpublished)
Malaysia	MN529663–MN529796	MN529797–MN529930	This study

## Appendix 4

**Table A4.** Frequency distribution of haplotypes according to localities. Highlighted columns indicate shared haplotypes.

Haplotype	Total	KS	KN	TW	SN	MH	PHI	TAI	HAI	IND
Hap_1	3	3								
Hap_2	1	1								
Hap_3	17	4	4	7		1				1
Hap_4	1	1								
Hap_5	21	5	6	1	5	3		1		
Hap_6	1	1								
Hap_7	1	1								
Hap_8	2	1	1							
Hap_9	1	1								
Hap_10	1	1								
Hap_11	1	1								
Hap_12	1	1								
Hap_13	1	1								
Hap_14	1	1								
Hap_15	1	1								
Hap_16	1	1								
Hap_17	1	1								
Hap_18	1	1								
Hap_19	1	1								
Hap_20	1	1								
Hap_21	1	1								
Hap_22	1	1								
Hap_23	1		1							
Hap_24	4		4							
Hap_25	1		1							
Hap_26	1		1							
Hap_27	1		1							
Hap_28	1		1							
Hap_29	1		1							
Hap_30	1		1							
Hap_31	1		1							
Hap_32	1		1							
Hap_33	1		1							
Hap_34	1		1							
Hap_35	1		1							
Hap_36	1		1							
Hap_37	1		1							
Hap_38	1		1							
Hap_39	1				1					
Hap_40	1				1					
Hap_41	5				5					
Hap_42	1				1					
Hap_43	1				1					
Hap_44	1				1					
Hap_45	2			1	1					
Hap_46	1				1					
Hap_47	1				1					
Hap_48	1				1					
Hap_49	1				1					
Hap_50	1				1					
Hap_51	1				1					
Hap_52	1				1					
Hap_53	1				1					
Hap_54	1				1					
Hap_55	1				1					
Hap_56	1				1					
Hap_57	1				1					
Hap_58	2					2				
Hap_59	1					1				
Hap_60	1					1				
Hap_61	1					1				
Hap_62	1					1				
Hap_63	1					1				
Hap_64	2					2				
Hap_65	1					1				
Hap_66	1					1				
Hap_67	1					1				
Hap_68	1					1				
Hap_69	2					2				
Hap_70	1					1				
Hap_71	1					1				
Hap_72	1			1						
Hap_73	2			2						
Hap_74	1			1						
Hap_75	1			1						
Hap_76	3			3						
Hap_77	1			1						
Hap_78	1			1						
Hap_79	1			1						
Hap_80	1			1						
Hap_81	1			1						
Hap_82	1			1						
Hap_83	1			1						
Hap_84	1								1	
Hap_85	1						1			
